# Society Meetings

**Published:** 1897-03

**Authors:** 


### Society Meetings.

THE Buffalo Academy of Medicine held meetings during the past month as follows:

*Section on Obstetrics and Gynecology.*—Tuesday evening, January 26, 1897, program: Résumé of fifty years of obstetrical practice, by Dr. John Hauenstein.

*Section on Surgery.*—Tuesday evening, February 2d, program: Results of 350 operative cases of inguinal hernia in the male, by Dr. William B. Coley, surgeon to the Hospital for Ruptured and Crippled, New York; Use of the galvano-cautery within the nose and throat, with demonstration, by Dr. Horace Clark.

*Section on Medicine.*—Tuesday evening, February 9th, program: Should the marriage contract be limited by law, by Dr. Rulison; Protective inoculations in typhoid fever and cholera, and the rôle of therapy in tetanus, tuberculosis, streptococcus infection and syphilis, by Dr. Frank J. Thornbury.

*Section on Pathology.*—Tuesday evening, February 16th, program: Cultivation of gonococci and exhibition of culture, by Dr. F. S. Busch; The workings of the Bureau of Animal Industry at East Buffalo, by N. P. Hinkley, D. V. M.; also, a special meeting of the whole Academy, with reference to pernicious legislation at Albany.

*Section on Obstetrics and Gynecology.*—Tuesday evening, February 23d, program : Drainage following abdominal operations, by Dr. Chauncey P. Smith ; Treatment of shock and collapse following operations, by Dr. John Parmenter.

---

THE New York Academy of Medicine celebrated its semi-centennial anniversary at the Carnegie Hall and at the Academy on Friday evening, January 29, 1897.

The President of the United States delivered the principal address, having come on from Washington at the instance of Dr. Joseph D. Bryant, president of the Academy, for that purpose. Dr. Bryant opened the exercises with a brief address, in which he referred succinctly to the purposes of the Academy as the representative body of the medical profession of the state of New York and its services to the municipality in the matter of many plans for the public health. Dr. Samuel S. Purple, one of the eight living founders of the Academy, followed with a brief address, in which he alluded to its early organisation and to the struggles incident thereto. Dr. Lewis A. Sayre, another of the founders, gave some interesting reminiscences of the early days of the Academy, and outlined some of its services for the health of the city, particularly in the matter of the establishment of quarantine. Dr. Abraham Jacobi, a former president of the Academy, was the formal orator of the evening. He outlined more at length the scope of the Academy, defining it the neutral ground of the profession, the only temple of safety and impartiality. He called special attention to the battle the Academy had made against quackery and begged the people to urge their representatives in legislative bodies to second their efforts in all possible ways. He touched upon the marvelous advance recently made in handling dangerous diseases, and declared that most of them had resulted from animal experimentations, for which he entered a strong and eloquent plea. The address of President Cleveland was well received, and after the exercises many of those present assembled at the Academy building, where a reception in honor of the president was held, followed by a collation.

